# The impact of Fisher's reproductive compensation on raising equilibrium frequencies of semidominant, nonlethal mutations under mutation/selection balance

**DOI:** 10.1093/g3journal/jkad231

**Published:** 2023-11-16

**Authors:** Ian M Hastings

**Affiliations:** Department of Tropical Disease Biology, Liverpool School of Tropical Medicine, Pembroke Place, Liverpool L3 5QA, UK

**Keywords:** reproductive compensation, mutation/selection balance, deleterious mutations

## Abstract

Fisher's reproductive compensation (fRC) occurs when a species’ demography means the death of an individual results in increased survival probability of his/her relatives, usually assumed to be full sibs. This likely occurs in many species, including humans. Several important recessive human genetic diseases cause early foetal/infant death allowing fRC to act on these mutations. The impact of fRC on these genetic conditions has been previously calculated and shown to be substantial as quantified by *ω*, the fold increase in equilibrium frequencies of the mutation under fRC compared with its absence, i.e. *ω* = 1.22 and *ω* = 1.33 for autosomal and sex-linked loci, respectively. However, the impact of fRC on the frequency of the much larger class of semidominant, nonlethal mutations is unknown. This is calculated here as *ω* = 2 − h*s for autosomal loci and *ω* up to 2 for sex-linked loci where *h* is dominance (varied between 0.05 and 0.95) and *s* is selection coefficient (varied between 0.05 and 0.9). These results show that the actions of fRC can almost double the equilibrium frequency of deleterious mutations with low values of *h* and/or *s* (noting that “low” is *s*∼0.05 to 0.1). It is noted that fRC may act differentially across the genome with genes expressed early in life being fully exposed to fRC while those expressed later in life may be unaffected; this could lead to systematic differences in deleterious allele frequency across the genome.

## Introduction

Population genetics as a discipline started in the 1930s and, at that time, was largely concerned with how advantageous mutations could spread through populations (positive selection) and how deleterious mutations could be eliminated (negative selection). The original assumptions underlying the calculations were simple: random mating of parental genotypes, nonoverlapping generations, and independent fates of parents, and offspring (e.g. Provine 1971; Charlesworth and Charlesworth 2010). This simple approach has been astonishingly successful and influential over the last 100 years and has been adapted to relax these assumptions such as allowing nonrandom mating through population substructuring, and investigating the effects of nonoverlapping generations, genetic substructure, and “inclusive fitness.” One relaxation, recognized by R.A. Fisher in the 1940s [as acknowledged by Race (1944)], was the realization that in some species (notably humans) the fates of offspring were not independent and the death of 1 individual could be compensated, to various degrees, by increased survival of its siblings. This occurred in humans before the demographic transition because females reproduced approximately every 4–5 years and early death of a fetus/infant allows the mother to reconceive earlier and produce a “replacement” sibling. The effect continued after the demographic transition because humans may choose a desired family size and “compensate” for any deaths by producing “replacement” siblings. The demographics of many other, nonhuman species also contain periods of intense intrabrood competition in which the death of an individual likely results in increased survival of his/her siblings [see Porcher and Lande (2005) for a recent application to plants which also considered the impact of Fisher's reproductive compensation (fRC) on the evolution of mating systems]. I term this effect fRC to distinguish it from a more recent form of “adaptive” reproductive compensation [i.e. facultatively increasing reproductive effort in animals mating with a suboptimal mate (e.g. Gowaty et al. 2007)]. The impact of fRC has been studied in relation to the obvious example of lethal human genetic diseases. In the case of an autosomal recessive lethal, the lethal *mm* genotype can only arise in matings between 2 carriers, i.e. both parents are +*m* where + represents the wildtype allele, and *m* is mutant, and this family consists of 25% *mm*, 50% +*m* and 25% ++ genotypes. In the absence of fRC the death of the *mm* genotype removes 2 *m* alleles from the population but if full fRC is operating a “replacement” sibling of genotype +*m* or ++ survives that has a 2/3 chance of carrying the mutant allele. Death of an *mm* individual therefore results in loss of 2 *m* alleles if fRC is absent but only 1.33 *m* alleles if fRC is present and fully compensating for the death of the *mm* genotype. As would be expected intuitively, fRC reduces selection against the *m* allele and the equilibrium frequency of the lethal mutations increases. Previous work has shown this increase is 22 and 33% for autosomal and sex-linked loci respectively (e.g. Templeton and Yokoyama 1980). The impact of fRC therefore has implications for the “genetic load” carried by a population. In the example of recessive lethals, it means an increased proportion of affected individuals are present in the population (at least at fertilization) although, interestingly, the impact of fRC helps reduce the demographic impact imposed on the population by this increased mutation frequency. This has social implications with some commentators speculating on how relaxed selection in modern human societies will translate into increased survival of genetically compromised individuals and hence into an increased genetic load, e.g. Crow (1997).

Investigations of the impact of reproductive compensation on recessive lethals alleles, both autosomal and sex-linked, have been developed over the last 70 years, although the same effect of fRC has been given different names such as “compensation,” “overcompensation” or “sibling replacement”; see for example Glass (1950), Li (1953), Lewontin (1953), Williams (1959), Hamilton (1966), Levin (1967), Feldman et al. (1969), Lange et al. (1978), Templeton and Yokoyama (1980), and Porcher and Lande (2005).

However, no such calculations appear to have been made for the much larger class of semidominant, nonlethal alleles, and this remains a significant knowledge gap. This manuscript describes how these calculations can be made and quantifies the impact of fRC in increasing the equilibrium frequency of such alleles.

## Methods

### Biological considerations and general modeling strategy

The nomenclature will denote “+” as the wildtype allele and “*m*” as the deleterious mutant allele, giving 3 autosomal genotypes ++, +*m*, and *mm*. In the case of sex-linked loci, males carry only a single allele plus the Y chromosome giving 3 female genotypes ++, +*m*, and *mm* and 2 male diploid genotypes +Y and *m*Y. As in conventional population genetics, the frequency of the wildtype allele is denoted *p*, and frequency of the mutant is *q*. Fitness of the ++ genotype is 1, fitness of +*m* is 1 − h*s, and of *mm* is 1 − *s* where *h* is dominance and *s* is the selection coefficient acting against the double mutant *mm* genotype. When considering sex-linked loci the fitness of the male +Y genotype is 1 and that of the *m*Y is 1 − *s*. The mutation is assumed to occur at the gamete stage and occurs from wildtype to mutant (“back mutation” from mutant to wildtype is assumed to be negligible).

Previous models of fRC considered recessive lethals (e.g. Templeton and Yokoyama 1980; Koeslag and Schach 1984; Hastings 2000) so only 2 adult autosomal genotypes were present, i.e. ++ and +*m*. fRC could therefore only operate in mating involving +*m* by +*m* genotypes where 25% of offspring would die and the “replacement” genotypes ++ and +*m* each had the fitness of 1 (because *h* = 0). The standard way to allow for fRC, in this case, was to calculate brood size, *B*, after fRC had occurred, as follows:


B=Z+(1−Z)*R

where *Z* is the proportion of the brood surviving genetic selection, and (1−*Z*) is the proportion dying. Setting *R* = 0 indicates that fRC is absent while setting *R* = 1 restores full brood size. In biological terms, a species whose demography causes the fates of offspring to be entirely independent would have *R* = 0, while a species whose demography generates intense intrabrood competition may have a value of *R* close to 1.

The problem when considering semidominant mutations is that different values of *R* may occur within different broods. For example, if *s* = 0.95 (so 95% of *mm* genotypes die) then in a *mm* by *mm* mating, 95% of the brood may die, and the ability of fRC to restore the full brood size may be implausible in this brood, but fRC may be capable of fully restoring brood size in a ++ by +*m* mating where a maximum of 50% of offspring may die. This could be addressed by using different *R* value for each mating type, e.g. *R* may be close to 1 in the mating of ++ with +*m* but may be much lower in the mating of *mm* with *mm* where most offspring may die. Here, an alternative, more explicit approach is developed based on the potential for “competitive release” within the brood [the phrase is borrowed from malaria intrahost dynamics (Wargo et al. 2007) which recognizes that death of some individuals frees up resources that allow increased survival of the remaining malaria parasites competing for those resources; the same phrase is used in ecology to describe competition between species rather than individuals]. Competitive release within broods can be expressed as


B=Z*C

where *C* is the competitive release coefficient. The baseline value of *C* = 1 means the competitive release is absent, a value of *C* = 2 states that survival of individuals in a reduced brood size can potentially double due to competitive release, and so on. Importantly, the size of brood after the actions of fRC cannot exceed the value of the brood produced by ++ by ++ mating whose value is set to 1, so *B* will be the lower of


B=Z*CorB=1 (1)

**Fig. 1.**
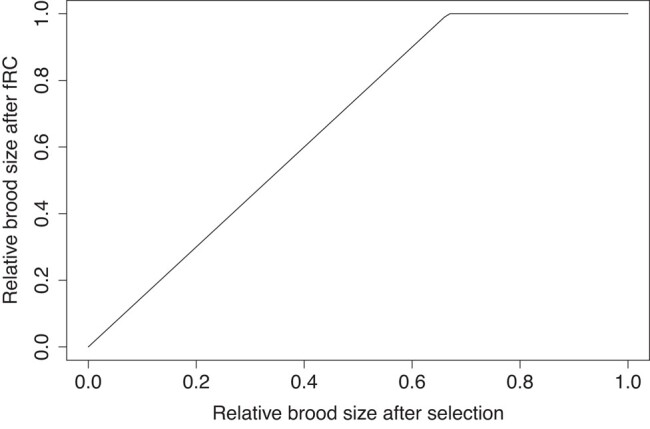
The effects of fRC on brood size after genetic selection. The competitive release coefficient is assumed to be 1.5 and the plot is obtained from Equation (1). Brood sizes after fRC are relative to a brood consisting solely of wildtype homozygotes (whose value is set to 1). In this example fRC can compensate for the loss of up to a third of the brood before its size starts to diminish, i.e. has a slope of 1.5 when the *X* axis value falls below 0.67.

An illustrative example for *C* = 1.5 is given on Fig. 1. Note that *Z* and *C* are independent of each other and of the genotype(s) surviving in the brood. For example, if *Z* = 0.1 (i.e. 10% of the brood survive) then that 10% will have increased survival, determined by *C*, irrespective of their genotype i.e. it is immaterial whether that 10% consists of wildtype or hetero/homozygous mutants or a mixture of each. An implicit assumption is, therefore, that selection occurs before fRC acts. More sophisticated quantifications of fRC can presumably be constructed to make the size of brood after fRC a more complex function of reduced brood size and genotype and used below in Equation (2), but the intention in this work is to establish the basic principles of fRC rather than provide a detailed examination of how competitive release operates.

It is assumed that fRC occurs within broods of full sibs. It is not necessary to specify the mechanism by which fRC occurs, simply that it exists (for example it may be that, in humans, mothers produce an extra offspring to compensate for a dead embryo/fetus/offspring, while in other species it may be intense competition within the brood for resources such as food that leads to fRC).

In standard theory, the frequencies of genotypes are tracked independently. However, under fRC the fates of genotypes differ depending on what type of brood they are born into, e.g. a +*m* genotype may occur in a brood consisting of all +*m* siblings (i.e. from a ++ by *mm* mating), or with a mixture of 50% +*m* and 50% ++ siblings (i.e. from a +*m* by ++ mating). It is, therefore, necessary to track mating between parental genotypes to recognize that broods differ in genetic composition [as in Koeslag and Schach (1984) although they only required 3 matings as *mm* genotypes were assumed lethal in their calculations].

The genetic calculations require a multistage computation for each of the 6 mating types, *i*, and 3 parameters are calculated for each mating type:

- The frequency of the mating type, denoted *M_i_*.
- The size of the brood after genetic selection and fRC [from Equation (1)], denoted *B_i_*.
- The proportion of each genotype surviving the brood to enter the breeding population for the next generation i.e. *P*(*++*)*_i_*, *P*(*+m*)*_i_*, and *P*(*mm*)*_i_*,.

Multiplying these 3 factors together generates the genotypes surviving from each mating type which can then be summed and normalized to obtain the parental genotypes of the next generation, i.e.


$$f^{\prime }(++)=\;\frac{\sum_{i=1}^{6}\;(M_{i}*B_{i}*P{\left(++\right)}_{i})}{N}$$ (2a)

$$f^{\prime }(+m)=\;\frac{\sum_{i=1}^{6}\;(M_{i}*B_{i}*P{\left(+m\right)}_{i})}{N}$$ (2b)

$$f^{\prime }(mm)=\;\frac{\sum_{i=1}^{6}\;(M_{i}*B_{i}*P{\left(mm\right)}_{i})}{N}$$ (2c)

where *N* is the normalizing factor equal to the numerators of Equations (2a)–(2c).

This describes the process for autosomal loci. An analogous process is used when investigating sex-linked loci except that it is necessary to track male and female genotypes separately (see *Sex-linked genes* below).

Mutation occurs in the brood genotypes, i.e. a proportion 2*µ* of ++ brood genotypes become +*m* and a proportion *µ* genotypes of type +*m* become *mm* where *µ* is the mutation rate from wildtype to mutant allele and we ignore back mutation from mutant to wildtype. Allowing mutation within broods allows for increased intrabrood genotype composition and hence competition and fRC. The alternative is to allow mutation at the parental stage (e.g. some ++ will mutate +*m*) but this underestimates intrabrood competition, e.g. a parent of genotype ++ producing all + gametes will produce a more homogenous brood that if that parent produced mostly + gametes with a small proportion of *m* gametes. It is doubtful if this makes any practical difference but is biologically more realistic as mutation in the germline means ++ parents will produce some *m* gametes.

The algorithms were constructed for autosomal and sex-linked loci as described below, then encoded as an R function and run to equilibrium using R v4.2.2 (R Core Team 2021).

### Autosomal loci

There are 3 genotypes and hence 6 different mating combinations. The values of *M_i_*, *B_i,_* and *P*()*_i_* can be obtained from each mating combination as given below. For clarity when calculating brood genotypes, the effect of mutation is presented in square brackets and the effect of selection in normal brackets.

*Mating type 1: ++* with *++*

Frequency of this mating types is *M*_1_ = *f*(++)^2^

Brood genotypes in absence of mutation: 100% ++

Frequencies of genotypes in broods after mutation and selection is


$$\;\;\;\;\;\;\;\;\;\;\;\;\;\;P(++{)}_{1}=[1-2\mu ]/N$$

$$P(+m{)}_{1}=[2\mu ]*(1-h*s)/N$$

$$\;\;\;\;\;\;\;\;\;\;\;\;\;\;\;\;\;\;\;\;\;\;\;\;\;\;\;\;\;\;\;\;\;\;\;\;\;\;\;\;\;P(mm{)}_{1}=0$$

where *N* is a normalizing factor equal to the sum of the numerators in the 3 equations above.

The size of the brood after selection is *Z*_1_ which is the sum of the numerators in the 3 equations above. The size of the brood after fRC is *B*_1_ = *Z*_1_*C or *B*_1_ = 1 whichever is the lower.

*Mating type 2: ++* with *+m*.

Frequency of this mating types is *M*_2_ = 2*f(++)*f(+*m*)

Brood genotypes in absence of mutations: 50% ++, 50% +*m*

Frequencies of genotypes in broods after mutation and selection is


$$\;\;\;\;\;\;\;\;\;\;\;\;\;\;\;\;\;\;\;\;\;\;\;\;\;\;\;\;\;\;\;\;\;\;\;\;\;\;\;\;\;\;\;\;\;P(++{)}_{2}=[0.5*(1-2\mu )]/N$$

$$P(+m{)}_{2}=[0.5*(1-\mu )+0.5*2\mu ]*(1-h*s)/N$$

$$\;\;\;\;\;\;\;\;\;\;\;\;\;\;\;\;\;\;\;\;\;\;\;\;\;\;\;\;\;\;\;\;\;\;\;\;\;\;\;\;\;\;\;\;\;\;\;\;\;\;\;\;\;\;\;\;P(mm{)}_{2}=[0.5\mu ]*(1-s)/N$$

where *N* is a normalizing factor equal to the sum of the numerators in the 3 equations above.

The size of the brood after selection is *Z*_2_ which is the sum of the numerators in the 3 equations above. Size of brood after fRC is *B*_2_ = *Z*_2_*C or *B*_2_ = 1 whichever is lower.

*Mating type 3: ++* with *mm*.

Frequency of this mating type is *M*_3_ = 2*f(++)*f(*mm*)

Brood genotypes in absence of mutation: 100% +*m*

Frequencies of genotypes in broods after mutation and selection is


$$\;\;\;\;\;\;\;\;\;\;\;\;\;\;\;\;\;\;\;\;\;\;\;\;\;\;\;\;\;\;\;\;\;\;\;\;\;\;\;\;\;\;\;\;\;\;P(++{)}_{3}=0$$

$$P(+m{)}_{3}=[1-\mu ]*(1-h*s)/N$$

$$\;\;\;\;\;\;\;\;\;\;\;\;\;\;\;\;\;\;P(mm{)}_{3}=[\mu ]*(1-s)/N$$

where *N* is a normalizing factor equal to the sum of the numerators in the 3 equations above.

The size of the brood after selection is *Z*_3_ which is the sum of the numerators in the 3 equations above. The size of the brood after fRC is *B*_3_ = *Z*_3_*C or *B*_3_ = 1 whichever is lower.

*Mating type 4: +m* with *+m*.

Frequency of this mating types is *M*_4_ = *f*(+*m*)^2^

Brood genotypes in the absence of mutation: 25% ++, 50% +*m*, 25% *mm*

Frequencies of genotypes in broods after mutation and selection is


$$\;\;\;\;\;\;\;\;\;\;\;\;\;\;\;\;\;\;\;\;\;\;\;\;\;\;\;\;\;\;\;\;\;\;\;\;\;\;\;\;\;\;\;\;\;P(++{)}_{4}=[0.25*(1-2u)]/N$$

$$P(+m{)}_{4}=[0.5*(1-\mu )+0.25*2\mu ]*(1-h*s)/N$$

$$\;\;\;\;\;\;\;\;\;\;\;\;\;\;\;\;\;\;\;\;\;\;\;\;\;\;\;\;\;\;\;\;\;\;P(mm{)}_{4}=[0.25+0.5\mu ]*(1-s)/N$$

where *N* is a normalizing factor equal to the sum of the numerators in the 3 equations above.

The size of the brood after selection is *Z*_4_ which is the sum of the numerators in the 3 equations above. The size of the brood after fRC is *B*_4_ = *Z*_4_*C or *B*_4_ = 1 whichever is lower.

*Mating type 5: +m* with *mm*.

Frequency of this mating types is *M*_5_ = 2*f(+*m*)*f(*mm*)

Brood genotypes in the absence of mutation: 50% +*m*, 50% *mm*

Proportion of genotypes in broods after mutation and selection is


$$\;\;\;\;\;\;\;\;\;\;\;\;\;\;\;\;\;\;\;\;\;\;\;\;\;\;\;\;\;\;\;\;\;\;\;\;\;\;\;\;\;\;\;\;\;\;\;\;\;\;\;\;\;\;\;\;\;\;\;\;\;P(++{)}_{5}=0$$

$$P(+m{)}_{5}=[0.5*(1-\mu )]*(1-h*s)/N$$

$$\;\;\;\;\;\;\;\;\;P(mm{)}_{5}=[0.5+0.5\mu ]*(1-s)/N$$

where *N* is a normalizing factor equal to the sum of the numerators in the 3 equations above.

The size of the brood after selection is *Z*_5_ which is the sum of the numerators in the 3 equations above. The size of the brood after fRC is *B*_5_ = *Z*_5_*C or *B*_5_ = 1 whichever is lower.

*Mating type 6: mm* with *mm*.

Frequency of this mating types is *M*_6_ = *f*(*mm*)^2^

Brood genotypes in absence of mutation: 100% *mm*

The proportion of genotypes in broods after mutation and selection is


$$\;\;\;\;\;\;\;\;\;\;\;\;\;\;\;\;\;\;\;\;\;\;\;\;\;\;P(++{)}_{6}=0$$

$$\;\;\;\;\;\;\;\;\;\;\;\;\;\;\;\;\;\;\;\;\;\;\;\;\;\;P(+m{)}_{6}=0$$

$$P(mm{)}_{2}=1*(1-s)/N$$

where *N* is a normalizing factor equal to the sum of the numerators in the 3 equations above.

The size of the brood after selection is *Z*_6_ = (1 − *s*). The size of the brood after fRC is *B*_6_ = *Z*_6_*C or *B*_6_ = 1 whichever is lower.

These values of *M_i_*, *B_i_*, and *P*()*_i_* obtained for each of the 6 mating types were then used in Equation (2) as described above, i.e. to obtain genotype frequencies at the end of each generation. Equilibrium frequencies at autosomal loci were obtained using the R function “uniiroot” as only a single variable has to be solved (i.e. equilibrium frequency of mutations). This gives equilibrium mutation frequency in adults, *q_a_* as


$$\hat{q_{a}}=f(+m)*0.5+f(mm)$$

Most population genetic analysis census the allele frequencies at the gamete stage (i.e. after mutation, before fertilization, and selection) so it is necessary to allow an extra episode of mutation which allows gametic mutation frequency, *q_g_*, can be calculated as


$$\hat{q_{g}}=\;q_{a}+(1-q_{a})\mu$$ (3)

This is the mutation frequency used in subsequent analyzes and replicates the standard results from the population genetic literature (see later).

### Sex-linked genes

We assume an *XY* system, i.e. males are the heterogametic sex. There are 5 diploid genotypes, i.e.


♂(+Y),♂(mY)

♀(++),♀(+m)♀(mm)

whose adult frequencies are denoted by the prefix *f*, i.e.


f♂(+Y),f♂(mY)

f♀(++),f♀(+m)f♀(mm)

There are 6 different mating combinations and, as for autosomal loci, the values of *M_i_*, *B_i,_* and *P*()*_i_* can be obtained from each mating combination as given below.

*Mating type 1: ♂(+Y)* with ♀*(++)*

Frequency of this mating types is *M*_1_ = *f♂*(*+Y*) * f♀(*++*)

Brood genotypes in the absence of mutation: 50% ♂(+*Y*), 50%♀(++)

The proportions of genotypes in broods after mutation and selection are


$$\;\;\;\;\;\;\;\;\;\;\;\;\;\;\;P(♂,+Y{)}_{1}=[0.5*(1-\mu )]/N$$

$$\;\;\;\;\;\;\;\;\;\;\;\;P(♂,mY{)}_{1}=[0.5\mu ]*(1-s)/N$$

$$\;\;\;\;\;\;\;\;\;\;\;\;P(♀,++{)}_{1}=[0.5*(1-2\mu )]/N$$

$$P(♀,+m{)}_{1}=[0.5*2u]*(1-h*s)/N$$

$$\;\;\;\;\;\;\;\;\;\;\;\;\;\;\;\;\;\;\;\;\;\;\;\;\;\;\;\;\;\;\;\;\;\;\;\;\;\;\;\;\;\;\;\;\;\;\;\;\;\;\;P(♀,mm{)}_{1}=0$$

where *N* is a normalizing factor equal to the sum of the numerators.

The size of the brood after selection is *Z*_1_ which is the sum of the numerators in the 5 equations above. The size of the brood after fRC is *B*_1_ = *Z*_1_*C or *B*_1_ = 1 whichever is lower.

*Mating type 2: ♂(+Y)* with *♀(+m)*

Frequency of this mating types is *M*_2_ = f♂(+Y) * f♀(+m)

Brood genotypes in absence of mutation: 25% ♂(+Y), 25% ♂(*m*Y), 25%♀(++), 25%♀(+*m*)

Proportions of genotypes in broods after mutation and selection are


$$\;\;\;\;\;\;\;\;\;\;\;\;\;\;\;\;\;\;\;\;\;\;\;\;\;\;\;\;\;\;\;\;\;\;\;\;\;\;\;\;\;\;\;\;\;\;\;\;\;\;\;\;\;\;\;\;\;P(♂,+Y{)}_{2}=[0.25*(1-\mu )]/N$$

$$\;\;\;\;\;\;\;\;\;\;\;\;\;\;\;\;\;\;\;\;\;\;\;\;\;\;\;\;\;\;\;\;\;P(♂,mY{)}_{2}=[0.25+0.25\mu ]*(1-s)/N$$

$$\;\;\;\;\;\;\;\;\;\;\;\;\;\;\;\;\;\;\;\;\;\;\;\;\;\;\;\;\;\;\;\;\;\;\;\;\;\;\;\;\;\;\;\;\;\;\;\;\;\;\;P(♀,++{)}_{2}=[0.25*(1-2\mu )]/N$$

$$P(♀,+m{)}_{2}=[0.25*(1-\mu )+0.25*2u]*(1-h*s)/N$$

$$\;\;\;\;\;\;\;\;\;\;\;\;\;\;\;\;\;\;\;\;\;\;\;\;\;\;\;\;\;\;\;\;\;\;\;\;\;\;\;\;\;\;\;\;\;\;\;\;\;\;\;P(♀,mm{)}_{2}=[0.25\mu ]*(1-s)/N$$

where *N* is a normalizing factor equal to the sum of the numerators.

The size of the brood after selection is *Z*_2_ which is the sum of the numerators in the 5 equations above. The size of the brood after fRC is *B*_2_ = *Z*_2_*C or *B*_2_ = 1 whichever is lower.

*Mating type 3: ♂(+Y)* with *♀(mm)*

Frequency of this mating types is *M*_3_ = f♂(+Y) * f♀(mm)

Brood genotypes in absence of mutation: 50% ♂(*m*Y), 50%♀(+*m*)

Proportions of genotypes in broods after mutation and selection are


$$P(♂,+Y{)}_{3}=0$$

$$\;\;\;\;\;\;\;\;\;\;\;\;\;\;\;\;\;\;\;\;\;\;\;\;\;\;\;P(♂,mY{)}_{3}=0.5*(1-s)/N$$

$$\;\;\;\;\;\;\;\;\;\;\;\;\;\;\;\;\;\;\;\;\;\;\;\;\;\;\;\;\;\;\;\;\;\;\;\;\;\;\;\;\;\;\;\;\;\;\;\;\;\;\;\;\;\;\;\;\;\;\;\;P(♀,++{)}_{3}=0$$

$$P(♀,+m{)}_{3}=[0.5*(1-\mu )]*(1-h*s)/N$$

$$\;\;\;\;\;\;\;\;\;\;\;\;\;\;\;\;\;\;\;\;\;\;P(♀,mm{)}_{3}=[0.5\mu ]*(1-s)/N$$

where *N* is a normalizing factor equal to the sum of the numerators.

The size of the brood after selection is *Z*_3_ which is the sum of the numerators in the 5 equations above. The size of the brood after fRC is *B*_3_ = *Z*_3_*C or *B*_3_ = 1 whichever is lower

*Mating type 4: ♂(mY)* with *♀(++)*

Frequency of this mating types is *M*_4_ = f♂(mY) * f♀(++)

Brood genotypes in absence of mutation: 50% ♂(+Y), 50%♀(+*m*)

Proportions of genotypes in broods after mutation and selection are


$$\;\;\;\;\;\;\;\;\;\;\;\;\;\;\;\;\;\;\;\;\;\;\;\;P(♂,+Y{)}_{4}=[0.5*(1-\mu )]/N$$

$$\;\;\;\;\;\;\;\;\;\;\;\;\;\;\;\;\;\;\;\;P(♂,mY{)}_{4}=[0.5\mu ]*(1-s)/N$$

$$\;\;\;\;\;\;\;\;\;\;\;\;\;\;\;\;\;\;\;\;\;\;\;\;\;\;\;\;\;\;\;\;\;\;\;\;\;\;\;\;\;\;\;\;\;\;\;\;\;\;\;\;\;\;\;\;\;\;\;\;P(♀,++{)}_{4}=0$$

$$P(♀,+m{)}_{4}=[0.5*(1-\mu )]*(1-h*s)/N$$

$$\;\;\;\;\;\;\;\;\;\;\;\;\;\;\;\;\;\;\;\;\;P(♀,mm{)}_{4}=[0.5\mu ]*(1-s)/N$$

where *N* is a normalizing factor equal to the sum of the numerators.

The size of brood the after selection is *Z*_4_ which is the sum of the numerators in the 5 equations above. The size of the brood after fRC is *B*_4_ = *Z*_1_*C or *B*_4_ = 1 whichever is lower

*Mating type 5: ♂(mY)* with *♀(+m)*

Frequency of this mating types is *M*_5_ = f♂(mY) * f♀(+m)

Brood genotypes in absence of mutation: 25% ♂(+Y), 25% ♂(*m*Y), 25%♀(+*m*), 25%♀(*mm*)

Proportions of genotypes in broods after mutation and selection are


$$\;\;\;\;\;\;\;\;\;\;\;\;\;\;\;\;\;\;\;\;\;\;\;\;P(♂,+Y{)}_{5}=[0.25*(1-\mu )]/N$$

$$P(♂,mY{)}_{5}=[0.25+0.25\mu ]*(1-s)/N$$

$$\;\;\;\;\;\;\;\;\;\;\;\;\;\;\;\;\;\;\;\;\;\;\;\;\;\;\;\;\;\;\;\;\;\;\;\;\;\;\;\;\;\;\;\;\;\;\;\;\;\;\;\;\;\;\;\;\;\;\;\;\;\;\;P(♀,++{)}_{5}=0$$

$$P(♀,+m{)}_{5}=[0.25*(1-\mu )]*(1-h*s)/N$$

$$\;\;\;P(♀,mm{)}_{5}=[0.25+0.25\mu ]*(1-s)/N$$

where *N* is a normalizing factor equal to the sum of the numerators.

The size of the brood after selection is *Z*_5_ which is the sum of the numerators in the 5 equations above. The size of the brood after fRC is *B*_5_ = *Z*_5_*C or *B*_5_ = 1 whichever is lower.

*Mating type 6: ♂(mY)* with *♀(mm)*

Frequency of this mating types is *M*_6_ = f♂(mY) * f♀(mm)

Brood genotypes in absence of mutation: 50% ♂(*m*Y), 50%♀(*mm*)

Proportions of genotypes in broods after mutation and selection are


$$\;\;\;\;\;\;\;\;\;\;\;\;\;\;\;\;\;\;\;\;\;\;\;\;\;\;\;\;\;\;\;\;\;\;\;\;P(♂,+Y{)}_{6}=0$$

$$P(♂,mY{)}_{6}=[0.5]*(1-s)/N$$

$$\;\;\;\;\;\;\;\;\;\;\;\;\;\;\;\;\;\;\;\;\;\;\;\;\;\;\;\;\;\;\;\;\;\;\;\;P(♀,++{)}_{6}=0$$

$$\;\;\;\;\;\;\;\;\;\;\;\;\;\;\;\;\;\;\;\;\;\;\;\;\;\;\;\;\;\;\;\;\;\;\;\;\;\;\;P(♀,+m{)}_{6}=0$$

$$P(♀,mm{)}_{6}=[0.5]*(1-s)/N$$

where *N* is a normalizing factor equal to the sum of the numerators.

The size of the brood after selection is *Z*_6_ which is the sum of the numerators in the 5 equations above. The size of the brood after fRC is *B*_6_ = *Z*_6_*C or *B*_6_ = 1 whichever is lower

The algorithm then proceeds as in Equation (2) but tracking female and male genotypes separately, i.e.

For males:


$$f^{\prime }♂(+Y)\;=\;\frac{\sum_{i=1}^{6}\;(M_{i}*B_{i}*\;P{\left(♂,+Y\right)}_{i})}{N_{m}}$$

$$f^{\prime }♂(mY)\;=\;\frac{\sum_{i=1}^{6}\;(M_{i}*B_{i}*\;P{\left(♂,mY\right)}_{i})}{N_{m}}$$

where *N_m_* is a normalizing factor equal to the sum of the 2 numerators in the male genotype equations.

For females:


$$f^{\prime }♀(++)\;=\;\frac{\sum_{i=1}^{6}\;(M_{i}*B_{i}*\;P{\left(♀,++\right)}_{i})}{N_{f}}$$

$$f^{\prime }♀(+m)\;=\;\frac{\sum_{i=1}^{6}\;(M_{i}*B_{i}*\;P{\left(♀,+m\right)}_{i})}{N_{f}}$$

$$f^{\prime }♀(mm)\;=\;\frac{\sum_{i=1}^{6}\;(M_{i}*B_{i}*\;P{\left(♀,mm\right)}_{i})}{N_{f}}$$

where *N_f_* is a normalizing factor equal to the sum of the 3 numerators in the female genotype equations.

Note that 2/3 of sex-linked alleles are in females, so overall frequencies of the mutant allele in the adult population, $\hat{q_{a}}$, is


$$\hat{q_{a}}=\frac{f♂(mY)}{3}+\frac{2*[f♀(+m)*0.5+♀(mm)]}{3}$$

and in the gametes is, as in Equation 3,


$$\hat{q_{g}}=\;q_{a}+(1-q_{a})\mu$$

Templeton and Yokoyama (1980) and Hastings (2000) defined $\hat{q}$ as the frequency of affected males (see also Crow (1986), his Equation 4.23 and discussion); since the mutation is assumed to be lethal in males, then the mutant alleles can only be transmitted through the female line so


$$\hat{q}=\frac{f♀(+m)}{2}+\mu$$

which is the definition used to check the method recovers the standard results given later in Equations (7) and (8).

Equilibrium allele frequencies were defined as occurring when mean frequency, $\hat{q}$ differed by less than a factor of 0.0000001 in consecutive iterations. The simulations were started with extremely low mutant frequencies (10^−9^), and extremely high frequencies (1–10^−9^), and the R scripts verified that both iterate onto the same equilibrium value of $\hat{q}$ (convergence to equilibrium is rapid for sex-linked loci so this transparent approach was preferred to using the uniroot function).

### Sex-linked loci with sexual dimorphism

I also investigated the impact of fRC on sex-linked genes in species that are sexually dimorphic to the extent that fRC can only occur within sexes, i.e. death of a male can only be compensated by increased survival of his brothers and not by increased survival of his sisters. Similarly for females, i.e. death of a female can only result in increased survival of her sisters. The methods used for the simulations were analogous to, and extremely similar with, methods used for sex-linked loci described in *Sex-linked genes*. The only difference is that fRC only occurs within each sex in the brood. The methods are therefore described in Supplementary File 1 to avoid repetition.

### Checking the algorithm recovers standard equations

Setting the competitive release coefficient, *C*, to 1 means fRC is absent and the algorithms should therefore give the standard results for mutation/selection balance given below. Alternatively, if fRC is set high (*C* = 100 is used here) then the algorithm should obtain the published results on recessive mutations obtained when fRC is sufficiently high that it allows full replacement in all brood types. These previous results are as follows:

*Autosomal loci:*


Forsemi-dominantmutations:q=μ/(h*s) (4)

$$\mathrm{For}\;\mathrm{recessive}\;\mathrm{mutations}:\;q=\sqrt{\mu /s}$$ (5)

$$\mathrm{For}\;\mathrm{recessive}\;\mathrm{lethal}\;\mathrm{mutations}\;\mathrm{under}\;\mathrm{full}\;\mathrm{fRC}:\;q=\sqrt{3\mu /2}\;$$ (6)

Equations (4) and (5) were taken from Charlesworth and Charlesworth (2010) their equations 4.3 and 4.4, and Equation (6) was taken from Templeton and Yokoyama (1980), noting that Koeslag and Schach (1984) and Hastings (2000) give numerically identical versions of Equation (6).

*Sex-linked loci:*


$$\mathrm{For}\;\mathrm{semi}\;\mathrm{dominant}\;\mathrm{mutations}:\;q=\frac{3\mu }{2hs+s}$$ (7)

Forrecessivelethals:q=3μ (8)

ForrecessivelethalsunderfullfRC:q=4μ (9)

Equation (7) was taken from Charlesworth and Charlesworth (2010) their equation 4.5. Equations (8) and (9) were taken from Templeton and Yokoyama (1980) their equations 4 and 7, and Hastings (2001) their equations 3 and 4. For simplicity, mutation rates were assumed to be 10^−7^.

### Running the simulations

All combinations of the following selection coefficients and dominance values were investigated, i.e.


s=0.05,0.1,0.15,0.2…0.9

h=0.05,0.1,0.15,0.2…0.95

and resulting equilibrium frequency of mutant alleles obtained as described above. Mutation rates were set to be 10^−5^, 10^−6^, 10^−7^, and 10^−9^ but did not affect the relative impact of fRC (see later) so a default of 10^−5^ was used.

## Results

### Autosomal loci

The algorithm for autosomal loci reliably recovered the standard, published results in the absence of fRC [Equations (4) and (5)], and the published result for recessive lethals when fRC is high [Equation (6)].

**Fig. 2.**
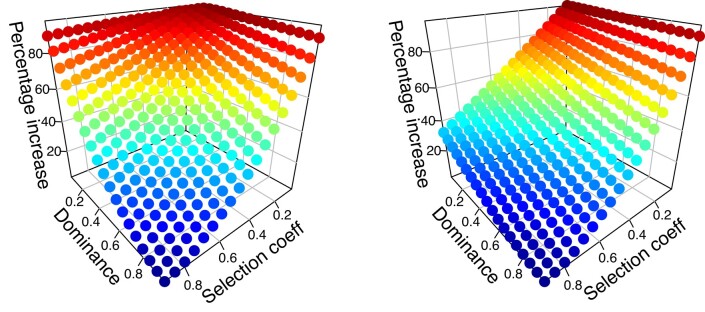
Percentage increase in equilibrium mutant allele frequency attributable to the actions of fRC, i.e. compared with standard result in the absence of fRC. Plots show results assuming the competitive release coefficient [Equation (1)], *C* = 1.5, and mutation rate is 10^−5^. The left panel shows results for autosomal loci and the right panel shows results for sex-linked loci.

**Fig. 3.**
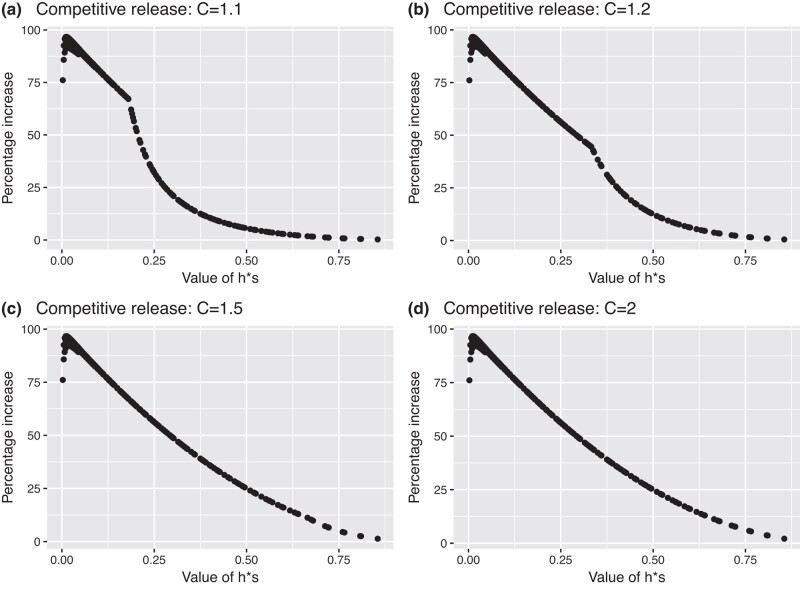
The increase in equilibrium mutant allele frequencies at autosomal loci attributable to fRC i.e. compared with standard theoretical results without fRC. The *X* axis is h*s (i.e. dominance multiplied by selection coefficient) under 4 illustrative values of competitive release coefficients [*C*; see Equation (1)]. The competitive release is unable to fully restore brood size once values of h*s exceed 0.18, 0.34, 0.66, and 1.0 for *C* = 1.1 (panel a), *C* = 1.2 (panel b), *C* = 1.5 (panel c), and *C* = 2 (panel d), respectively; see Equation (14).

Figure 2 shows the increase in equilibrium frequency under fRC compared with equilibrium frequency in its absence, as a function of dominance and selection coefficients. The surface is roughly symmetrical against the dominance and selection which is consistent with expectations as the low equilibrium frequencies of the mutations ensure most mutations will be in the heterozygous form whose fitness is 1 − h*s. The increases were therefore replotted against the value of h*s as shown on Fig. 3 for a range of competitive release coefficients. The increase is essentially linear against h*s until fRC breaks down and the increase falls relatively rapidly thereafter. The regression coefficients were calculated on the linear portions of the 4 panels of Fig. 3 and had an intercept of 100, with a slope of −100 in all cases, suggesting that the equilibrium frequency in the presence of full fRC can be obtained by updating Equation (4) to


$$q=\frac{u}{h*s}(2-h*s)$$ (10)

The result can be recovered algebraically by noting that when the frequency of mutations is low, most mutant alleles will be in the autosomal mating type 2: i.e. ++ with *+m* whose brood genotypes are 50% ++ and 50% +m. The fitness of the +m genotypes *w_+m_* in the absence of fRC is, therefore,


$$w_{+m}=1-hs$$

Rising to


$$w_{+m}=1-hs+\left(hs*\frac{1-hs}{1-hs+1}\right)$$

In the presence of fRC. The term in brackets is the number of replacements (“h*s” of them) multiplied by the proportion that is of genotype *+m* (the second factor in the brackets). Moving the brackets and simplifying slightly gives


$$w_{+m}=1-\left(hs-hs*\frac{1-hs}{2-hs}\right)=1-hs\left(1-\frac{1-hs}{2-hs}\right)=\;1-hs*\left(\frac{1}{2-hs}\right)$$

[The last step was obtaining using the algebraic rule that 1 − *a*/*b* = (*b* − *a*)/*b* which simplifies the expression in brackets to 1/(2 − *hs*)].

This suggests the equilibrium frequency of deleterious mutations under full fRC is


$$q=\frac{\mu }{h*s*\left(\frac{1}{2-h*s}\right)}=\;\frac{\mu }{h*s}(2-h*s)$$ (11)

Which is identical to Equation (10) obtained empirically by regression. It holds numerically over the approximate range h*s = 0.05–0.95 provided full fRC occurs (Fig. 3d). This relationship breaks down as h*s becomes very small as selection is then virtually absent, equilibrium frequency rises so the assumption that all selection occurs in autosomal mating type 2 is violated.

The equilibrium frequency in the absence of fRC is *u*/(h*s) [Equation (4)] revealing that that the fold increase in equilibrium frequencies driven by fRC, is


ω=(2−h*s) (12)

Equations (10) and (11) hold providing the competitive release coefficient [*C*; see Equation (1)] is sufficiently large to replace the proportion of individuals lost to genetic selection. The critical fraction of the brood, *F*, which can die and be replaced without a reduction in brood size can be calculated as


(1−F)*C=1

so


F=1−1/C (13)

which gives values of *F* as 0.09, 0.17, 0.33, and 0.5 for *C* = 1.1, 1.2, 1.5, and 2, respectively. We would therefore expect linearity to break down in Fig. 3 when competitive release is unable to replace all dead brood members. Provided equilibrium mutant allele frequency is sufficiently low that the proportion of double mutant genotypes is negligible, then most offspring with mutant alleles will be in matings ++ by *+m* in which case 50% will be *+m* so mortality in the brood will be 0.5*h*s. Linearity should therefore break down when this mortality exceeds *F*, i.e. when


0.5*h*s>Forh*s>2F (14)

which appears to be the case; see Fig. 3.

### Sex-linked loci

The method recovered all but one of the standard algebraic results already in the literature [i.e. Equations (7)–(9)], the exception being Equation (7), i.e. the equilibrium frequency of sex-linked semidominant mutations in the absence of fRC; this is explicable as a result of the algebraic assumptions made in derivation of Equation (7) as is explained in Supplementary File 2.

**Fig. 4.**
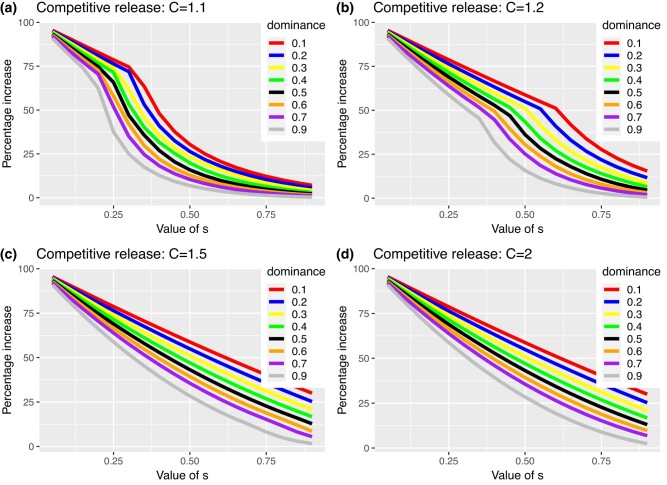
As for Fig. 3 but for sex-linked loci. Note that in this case of sex-linkage, it is necessary to disaggregate dominance (*h*) and selection coefficient (*s*) and is not possible to predict values of h*s where competitive release is unable to fully restore brood size.

Figure 2b provides an example of how fRC increases the equilibrium frequency of sex-linked deleterious mutations over a wide parameter range. The increase in mutant allele frequency compared with the standard result (i.e. in the absence of fRC) is far more dependent on the selection coefficient than on dominance (compared with autosomal loci). This most likely arises because most selection occurs in males who have only a single copy of the gene meaning dominance has no impact on selection in males. This complicates the plots of the increase against the magnitude of *h* and *s* shown on Fig. 4, compared with the autosomal loci shown in Fig. 3, but the same underlying patterns can be discerned, i.e. Fig. 4 shows a steady decline in percentage increase in mutation equilibrium frequency with *h* and *s*. This increase drops rapidly in Fig. 4a and b as competitive release becomes unable to compensate fully for the dead genotypes, with this drop being unnoticeable when its coefficient, *C* is 1.5 or 2 (in both Figs. 3 and 4) as competitive release is then sufficient to fully compensate for the dead genotypes. The key point from both Figs. 3 and 4 is that as h and s becomes small, then the equilibrium frequencies may be double that predicted in the absence of fRC.

### Sex-linked loci with sexual dimorphism

The results are presented and discussed in more detail in Supplementary File 1 but the basic result is that equilibrium allele frequencies can be up to 2.8-fold higher than predicted under standard theory (i.e. in the absence of fRC) when dominance is high, and selection coefficients are low (Supplementary Figs. 1.1 and 1.2 in Supplementary File 1).

## Discussion

The first thing to note is that this work is not directed toward specific mutations (in contrast to previous work that usually investigates recessive lethal mutations of importance to human health), rather it quantifies the extent to which life history and demographic traits can result in fRC altering a species genetics and, in particular, the equilibrium frequencies of deleterious mutations. It also establishes a general approach that can recover previous results on mutation/selection balance with, or without, fRC. It lacks algebraic solutions and requires numerical solutions when applied to sex-linked loci, but it is very flexible and can incorporate factors such as inbreeding coefficients into mating frequencies [previous work has shown inbreeding interacts with fRC, e.g. (Porcher and Lande 2005)] and can potentially include more sophisticated descriptions of fRC compared with the simple Equation (1) and Fig. 1.

One innovation developed above was to define and use a competitive release coefficient to quantify the extent of fRC [previous models of fRC assumed compensation was fully effective, i.e. all brood sizes remained unaffected by genetic deaths with the only exception (Koeslag and Schach 1984) allowing intermediate values of fRC, but were applied equally to all mating types]. The use of a competitive release coefficient is more biologically realistic and clarifies the dynamics behind fRC (e.g. Fig. 3) which can be explained as the combination of 2 effects. The first effect is the ability of fRC to compensate for the genetic deaths and maintain the brood size; when the limit of that ability is reached, competitive release becomes unable to restore full brood size (cf Fig. 1), and the breakpoints apparent in the panels of Fig. 3 occur. The second effect is that the relationship between the percentage increase in the mutation frequency and h*s is linear before the breakpoint. Recall that because equilibrium frequency of the mutant is low, the vast majority of the mutations occur in heterozygous form within broods of mainly wildtype genotypes (i.e. in ++ by *+m* matings). The magnitude of h*s determines the relative fitness between the mutant heterozygote and their wildtype siblings in these matings. As the fitness differences increase, the dead heterozygotes are increasingly replaced by their ++ siblings, hence overall transmission of the mutant allele from the brood falls as h*s increases.

The analogous methodology was used to investigate the impact of fRC on sex-linked loci when there is complete sexual dimorphism in the life-cycle stages where the fRC is acting, i.e. where a dead individual can only be replaced by a survivor of his/her own sex. This was performed mainly for curiosity and theoretical completeness as I know of no organisms with such strong dimorphism (although this undoubtably reflects my limited knowledge). It adds to our intuitive understanding of the actions and consequences of fRC and the basic result is that fRC in fully sexually dimorphic species may raise equilibrium frequencies of deleterious mutations up to 2.8-fold higher than would occur in the absence of fRC.

In these calculations, it was assumed that the fitness penalties *only* occur during the life-cycle stage(s) in which fRC is acting whereas, in reality, fitness penalties may extend throughout the life. As Porcher and Lande (2005) noted in their recent study of fRC and plant mating systems “Much embryo mortality is attributable to early acting, highly deleterious mutations (lethals and semilethals), whereas mildly deleterious mutations tend to act late in development during growth.” The calculations above evaluate semilethal mutations (generally defined as *s* > 0.5, *s*≠1) and the pattern of gene expression and demography of the organism determine the extent to which selection only occurs in life-cycle stages subject to fRC The assumption certainly applies to some mutations (i.e. those whose gene expression only occurs early in structured life cycles (such as larvae, pupae, tadpoles, and caterpillars) during which fRC may occur. The other, slightly circular argument is that the presence of fRC implies intense selection acting in early stages and this may dominate selection pressures acting against the same deleterious mutation in later life. One strategy would be to partition fitness costs between the fRC “brood” and post-fRC “selection” stages and apply them to both periods. However, the primary purpose of the work was to investigate and close a theoretical gap in quantifying how fRC may potentially affect semidominant, nonlethals, and present the current work as the limiting case that all selection pressure is occurring in the period when fRC is acting.

fRC appears to be a neglected process in evolution whose impact may explain many evolutionary features, not simply increased deleterious allele frequency. This paper has demonstrated computational ways to quantify this impact. In particular, the parameters of mutation rate, selection coefficient, dominance, and equilibrium frequencies are all linked in the equations predicting equilibrium mutation frequencies that occur at mutation/selection balance [see Equations (4)–(9) above] which are useful as estimates of 3 factors allow the 4th to be estimated. The actions of fRC mean that application of these equations may introduce up to 2-fold error in calculations. For example, estimating a mutation rate for a deleterious allele and then measuring its frequency (assumed to be at equilibrium) generates an estimate for h*s, i.e. selection acting on the heterozygote [by re-arrangement of Equation (4)]: this estimate of h*s would only apply if fRC was absent and would be up to 2-fold too low if high levels of fRC occur in the study species.

Finally, it is important to note that it is not simply a dichotomy between species that do, or do not, allow opportunities for fRC to occur. Probably more important than the dichotomy between species is the dichotomy between genes *within* a species whose demography allows fRC to occur. There will be 2 types of genes in such organisms, i.e. those genes whose expression occurs when fRC is acting and hence is affected by fRC, and those genes whose expression only occurs in life-cycle stages after fRC has occurred; the former will, all other factors being equal, have higher levels of deleterious variation than the second group. For instance, there will be differences in humans between genes whose effects occur early in development (which may be subject to fRC) and genes whose effects occur later after fRC has occurred (such as adult haemoglobins and adult-specific tissues such as eyes and ears). Bioinformatic approaches often compare genes within the same organism and the realization that they may be subject to slightly different selective forces may become important as differences in equilibrium frequencies may be attributed to difference in the magnitude of selection while, in principle, selection pressure acting on the genes may be the same, it is fRC that is driving the intragenomic differences in levels of deleterious genetic variation.

## Supplementary Material

jkad231_Supplementary_Data

## Data Availability

The R functions used to calculate equilibrium frequencies at mutation/selection balance under fRC were written by myself and are publicly available at https://github.com/ian-hastings/Fishers-Reproductive-compensation. Supplemental material available at G3 online.
