# Supplementary material for: The impact of Fisher's reproductive compensation on raising equilibrium frequencies of semidominant, nonlethal mutations under mutation/selection balance
Source: G3 (Bethesda). 2023 Nov 16;14(1):jkad231. doi: 10.1093/g3journal/jkad231 (PMC10755198; doi:10.1093/g3journal/jkad231)

**Supplemental File S1. Simulating the impact of fRC in species with complete sexual dimorphism.**

**1. Introduction.**

This Supplemental File describes the impact of fRC on sex-linked genes in species that are sexually dimorphic to the extent that fRC can only occurs within sexes i.e. death of a male can only be compensated by increased survival of his brothers and not by increased survival of his sisters. Similarly for females i.e. death of female can only result in increased survival of her sister.

This was for intellectual curiosity, theoretical completeness, and to ensure the impact of fRC had been fully explored and was explicable across several demographic scenarios.

**2. Methods.**

The methodology is conceptually identical to the sex-linked calculations described in the main text i.e. assuming fRC acts of the whole brood. The only significant difference when considering sexual dimorphism is that fRC has to be calculated independently for each sex (so the maximum brood size of each sex is 0.5). The following description parallels that for sex-linked loci in the main text.

*Mating type 1: ♂(+Y) with ♀(++)*

Frequency of this mating types is M_1_= f*♂(+Y) * f♀(++)*

Brood genotypes in absence of mutation: 50% ♂(+Y), 50%♀(++)

Proportions of genotypes in broods after mutation and selection are

$${P\left( ♂,+Y \right)}_{1}=[0.5*(1-\mu)]/N_{m}$$

$${P\left( ♂,mY \right)}_{1}=[0.5\mu]*(1-s)/N_{m}$$

$${P\left( ♀,++ \right)}_{1}=[0.5*(1-2\mu)]/N_{f}$$

$${P\left( ♀,+m \right)}_{1}=[0.5*2u]*(1-h*s)/N_{f}$$

$${P\left( ♀,mm \right)}_{1}=0$$

Where:

*N_m_* is a normalising factor for males, equal to the sum of the numerators of the two male equations above.

*N_f_* is a normalising factor for females, equal to the sum of the numerators of the three female equations above.

Size of male brood after selection is $Z_{1}^{m}$ which is the sum of the numerators in the two male equations above. Size of male brood after fRC is $B_{1}^{m}=Z_{1}^{m}*C$ or $B_{1}^{m}$=0.5 whichever is the lower.

Size of female brood after selection is $Z_{1}^{f}$ which is the sum of the numerators in the three female equations above. Size of female brood after fRC is $B_{1}^{f}=Z_{1}^{f}*C$ or $B_{1}^{f}$=0.5 whichever is the lower.

*Mating type 2: ♂(+Y) with ♀(+m)*

Frequency of this mating types is M_2_= f*♂(+Y) * f♀(+m)*

Brood genotypes in absence of mutation: 25% ♂(+Y), 25% ♂(*m*Y), 25%♀(++), 25%♀(+*m*)

Proportions of genotypes in broods after mutation and selection are${P\left( ♂,+Y \right)}_{2}=[0.25*(1-\mu)]/N_{m}$

$${P\left( ♂,mY \right)}_{2}=[0.25+0.25\mu]*(1-s)/N_{m}$$

$${P\left( ♀,++ \right)}_{2}=[0.25*(1-2\mu)]/N_{f}$$

$${P\left( ♀,+m \right)}_{2}=[0.25*(1-\mu)+0.25*2u]*(1-h*s)/N_{f}$$

$${P\left( ♀,mm \right)}_{2}=[0.25\mu]*(1-s)/N_{f}$$

Where:

*N_m_* is a normalising factor for males, equal to the sum of the numerators of the two male equations above.

*N_f_* is a normalising factor for females, equal to the sum of the numerators of the three female equations above.

Size of male brood after selection is $Z_{2}^{m}$ which is the sum of the numerators in the two male equations above. Size of male brood after fRC is $B_{2}^{m}=Z_{2}^{m}*C$ or $B_{2}^{m}$=0.5 whichever is the lower.

Size of female brood after selection is $Z_{1}^{f}$ which is the sum of the numerators in the three female equations above. Size of female brood after fRC is $B_{2}^{f}=Z_{2}^{f}*C$ or $B_{2}^{f}$=0.5 whichever is the lower.

*Mating type 3: ♂(+Y) with ♀(mm)*

Frequency of this mating types is M_3_= f*♂(+Y) * f♀(mm)*

Brood genotypes in absence of mutation: 50% ♂(*m*Y), 50%♀(+*m*)

Proportions of genotypes in broods after mutation and selection are

$${P\left( ♂,+Y \right)}_{3}=0$$

$${P\left( ♂,mY \right)}_{3}=0.5*(1-s)/N_{m}$$

$${P\left( ♀,++ \right)}_{3}=0$$

$${P\left( ♀,+m \right)}_{3}=[0.5*(1-\mu)]*(1-h*s)/N_{f}$$

$${P\left( ♀,mm \right)}_{3}=[0.5\mu]*(1-s)/N_{f}$$

Where:

*N_m_* is a normalising factor for males, equal to the sum of the numerators of the two male equations above.

*N_f_* is a normalising factor for females, equal to the sum of the numerators of the three female equations above.

Size of male brood after selection is $Z_{3}^{m}$ which is the sum of the numerators in the two male equations above. Size of male brood after fRC is $B_{3}^{m}=Z_{3}^{m}*C$ or $B_{3}^{m}$=0.5 whichever is the lower.

Size of female brood after selection is $Z_{3}^{f}$ which is the sum of the numerators in the three female equations above. Size of female brood after fRC is $B_{3}^{f}=Z_{3}^{f}*C$ or $B_{3}^{f}$=0.5 whichever is the lower.

*Mating type 4: ♂(mY) with ♀(++)*

Frequency of this mating types is M_4_= f*♂(mY) * f♀(++)*

Brood genotypes in absence of mutation: 50% ♂(+Y), 50%♀(+*m*)

Proportions of genotypes in broods after mutation and selection are

$${P\left( ♂,+Y \right)}_{4}=[0.5*(1-\mu)]/N_{m}$$

$${P\left( ♂,mY \right)}_{4}=[0.5\mu]*(1-s)/N_{m}$$

$${P\left( ♀,++ \right)}_{4}=0$$

$${P\left( ♀,+m \right)}_{4}=[0.5*(1-\mu)]*(1-h*s)/N_{f}$$

$${P\left( ♀,mm \right)}_{4}=[0.5\mu]*(1-s)/N_{f}$$

Where:

*N_m_* is a normalising factor for males, equal to the sum of the numerators of the two male equations above.

*N_f_* is a normalising factor for females, equal to the sum of the numerators of the three female equations above.

Size of male brood after selection is $Z_{4}^{m}$ which is the sum of the numerators in the two male equations above. Size of male brood after fRC is $B_{4}^{m}=Z_{4}^{m}*C$ or $B_{4}^{m}$=0.5 whichever is the lower.

Size of female brood after selection is $Z_{4}^{f}$ which is the sum of the numerators in the three female equations above. Size of female brood after fRC is $B_{4}^{f}=Z_{4}^{f}*C$ or $B_{4}^{f}$=0.5 whichever is the lower.

*Mating type 5: ♂(mY) with ♀(+m)*

Frequency of this mating types is M_5_= f*♂(mY) * f♀(+m)*

Brood genotypes in absence of mutation: 25% ♂(+Y), 25% ♂(*m*Y), 25%♀(+*m*), 25%♀(*mm*)

Proportions of genotypes in broods after mutation and selection are

$${P\left( ♂,+Y \right)}_{5}=[0.25*(1-\mu)]/N_{m}$$

$${P\left( ♂,mY \right)}_{5}=[0.25+0.25\mu]*(1-s)/N_{m}$$

$${P\left( ♀,++ \right)}_{5}=0$$

$${P\left( ♀,+m \right)}_{5}=[0.25*(1-\mu)]*(1-h*s)/N_{f}$$

$${P\left( ♀,mm \right)}_{5}=[0.25+0.25\mu]*(1-s)/N_{f}$$

Where:

*N_m_* is a normalising factor for males, equal to the sum of the numerators of the two male equations above.

*N_f_* is a normalising factor for females, equal to the sum of the numerators of the three female equations above.

Size of male brood after selection is $Z_{5}^{m}$ which is the sum of the numerators in the two male equations above. Size of male brood after fRC is $B_{5}^{m}=Z_{5}^{m}*C$ or $B_{5}^{m}$=0.5 whichever is the lower.

Size of female brood after selection is $Z_{5}^{f}$ which is the sum of the numerators in the three female equations above. Size of female brood after fRC is $B_{5}^{f}=Z_{5}^{f}*C$ or $B_{5}^{f}$=0.5 whichever is the lower.

*Mating type 6: ♂(mY) with ♀(mm)*

Frequency of this mating types is M_6_= f*♂(mY) * f♀(mm)*

Brood genotypes in absence of mutation: 50% ♂(*m*Y), 50%♀(*mm*)

Proportions of genotypes in broods after mutation and selection are${P\left( ♂,+Y \right)}_{6}=0$

$${P\left( ♂,mY \right)}_{6}=[0.5]*(1-s)/N_{m}$$

$${P\left( ♀,++ \right)}_{6}=0$$

$${P\left( ♀,+m \right)}_{6}=0$$

$${P\left( ♀,mm \right)}_{6}=[0.5]*(1-s)/N_{f}$$

Where:

*N_m_* is a normalising factor for males, equal to the sum of the numerators of the two male equations above.

*N_f_* is a normalising factor for females, equal to the sum of the numerators of the three female equations above.

Size of male brood after selection is $Z_{6}^{m}$ which is the sum of the numerators in the two male equations above. Size of male brood after fRC is $B_{z}^{m}=Z_{z}^{m}*C$ or $B_{z}^{m}$=0.5 whichever is the lower.

Size of female brood after selection is $Z_{6}^{f}$ which is the sum of the numerators in the three female equations above. Size of female brood after fRC is $B_{6}^{f}=Z_{6}^{f}*C$ or $B_{6}^{f}$=0.5 whichever is the lower.

The production of adult parents of next generation is as for sex-linked mutations without sex-specific fRC (Main text) except the values of $B_{i}$ are now sex specific i.e. $B_{i}^{m}$ or $B_{i}^{f}$

For males

$$f'♂(+Y) = \frac{\sum_{i=1}^{6} \left( M_{i}*{B_{i}^{m}}* {P\left( ♂,+Y \right)}_{i} \right)}{N_{m}}$$

$$f'♂(+Y) = \frac{\sum_{i=1}^{6} \left( M_{i}*B_{i}^{m}* {P\left( ♂,+Y \right)}_{i} \right)}{N_{m}}$$

where N_m_ is a normalising factor equal to the sum of the two numerators in the male genotype equations.

For females

$$f'♀(++) = \frac{\sum_{i=1}^{6} \left( M_{i}*B_{i}^{f}* {P\left( ♀,++ \right)}_{i} \right)}{N_{f}}$$

$$f'♀(+m) = \frac{\sum_{i=1}^{6} \left( M_{i}*B_{i}^{f}* {P\left( ♀,+m \right)}_{i} \right)}{N_{f}}$$

$$f'♀(mm) = \frac{\sum_{i=1}^{6} \left( M_{i}*B_{i}^{f}* {P\left( ♀,mm \right)}_{i} \right)}{N_{f}}$$

where N_f_ is a normalising factor equal to the sum of the three numerators in the female genotype equations.

**3. Results.**

When fRC is absent (obtained by setting C=1) the algorithm for sexual dimorphism gives identical results to those obtained for sex-linked loci without polymorphism and recovers previous published results (i.e. Equations 7 and 8 of main text). The case of C=1 generates the allele frequencies that occur in the absence of fRC so serve as the baseline scenario of non-fRC against which the impact of fRC is assessed.

Fig. S1.1 is analogous to the results for autosomal and sex-linked loci given on Fig. 2 of the main text; note the difference in the Y axis scale which, under sexual dimorphism is from 0 to 200% rather than from 0 to 100% on Fig. 2 of the main text i.e. sexual dimorphism can potentially lead to fRC almost tripling equilibrium mutant allele frequencies. The relationship with *h* and *s* is given on Fig. S1.2.

To understand the dynamics shown on Fig. S1.1, recall that when the mutant allele is very rare most selection occurs in the following two mating types

Type 2: ♂(+Y) with ♀(+*m*)

Brood genotypes: 25% ♂(+Y), 25% ♂(*m*Y), 25%♀(++), 25%♀(+*m*)

Type 4: ♂(*m*Y) with ♀(++)

Brood genotypes: 50% ♂(+Y), 50%♀(+*m*)

The pattern shown on Fig. S1.1 is complicated and is best explained by considering the two cases of high and low dominance.

*(i) When dominance is low* (i.e. the mutation is largely recessive), most selection falls on the ♂ (*m*Y) male offspring which are only present in mating #2. Their death is largely compensated by their ♂(+Y) brothers i.e. removal of a mutant allele is usually compensated by increased survival of wildtype alleles. This makes fRC much less effective at raising equilibrium frequency of mutations compared to the absence of dimorphism (because, in the latter, death of ♂(*m*Y) in mating type #2 are partially compensated by increased survival of their ♀ (+*m*) sisters). Within this region of low dominance, selection acts as follows:

- At high values of *s*, most surviving brothers in the brood are ♂(+Y) so the loss of mutant alleles is almost entirely compensated by wildtype alleles, meaning fRC has little effect when the mutant has high selection coefficient and low dominance (bottom left hand corner of Fig. S1.1 panels A and B).
- As the selection coefficient decreases, the proportion of ♂(*m*Y) among the surviving brothers increases so the impact of fRC increases i.e. death of mutant alleles tends to be compensated by survival of other mutant alleles in ♂(*m*Y) brothers; the impact of fRC consequently increases, and equilibrium frequency increases over the predictions of standard theory as shown along the left hand edge of Figs. S1.1(A) and S1.1(B).

*(ii) As dominance increases*, more selection falls on the female ♀(+*m*) genotypes which occur in mating types 2 and 3. These female genotypes in mating type 2 are mostly compensated by + alleles from replacement ++ sisters which reducing the impact of fRC in this mating type. However, the impact of fRC in mating #4 will be much larger because only ♀(+*m*) are present among the female offspring, so their deaths are always compensated by survival of the same genotype and selection against the mutant allele in females in this brood is effectively absent until fRC can no longer fully compensate for these female deaths. When C=1.5 full compensation can occur until >33% of the female brood die (Fig. 1 of main text) so the lines representing s= 0.5, 0.6, 0.8 and 0.9 in Fig.S1.1(B) shows a declining impact of fRC once *h*s* is greater than 33% i.e. when *h* exceeds 0.67, 0.56, 0.42, 0.37 respectively for these values of s.

In summary, fRC within sexual dimorphism can potentially increase equilibrium frequency above standard theory to a much greater extent than autosomal loci or sex-linkage without sexual dimorphism i.e., generating a maximum increase of ω≈2.8 compared of a maximum increase of ω≈2 in the other two scenarios.

**Fig. S1.1**. Percentage increase of equilibrium mutant allele frequency attributable to fRC acting on sex-linked loci in the presence of complete sexual dimorphism (i.e., compared to frequency in absence of fRC). Panel (A) shows the relationship with selection coefficient and dominance, assuming C=1.5 and mutation rate is 10^-5^. Panel (B) shows transects across the plot shown in Panel (A)


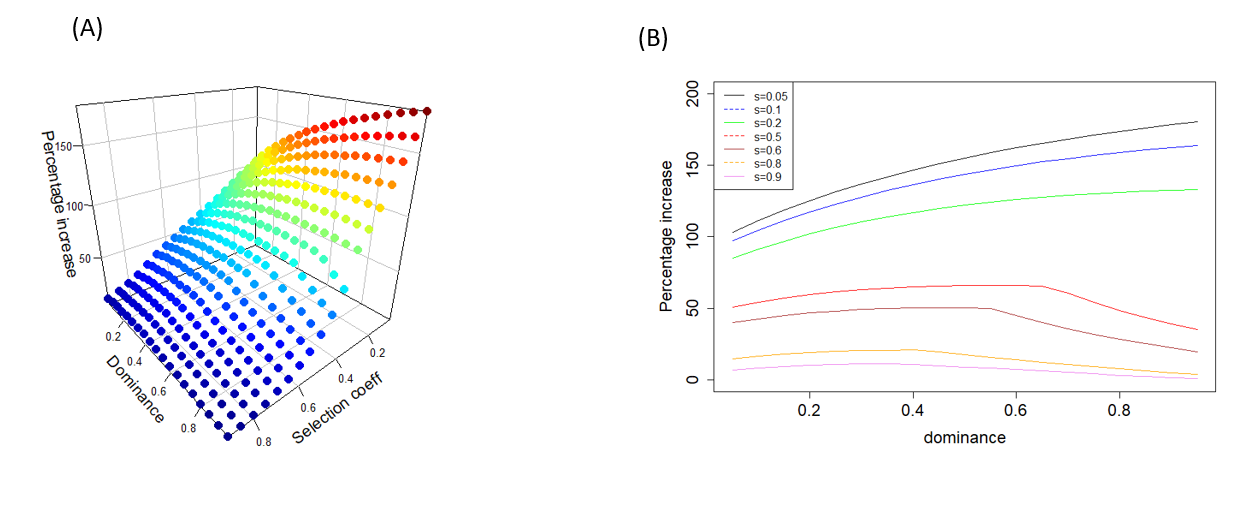


**Fig. S1.2**. Panels (A) and (B) are as for Fig. 3 of the main text but for sex-linked loci with fRC acting within sexual dimorphism with values for C=1.2 and C=1.5.


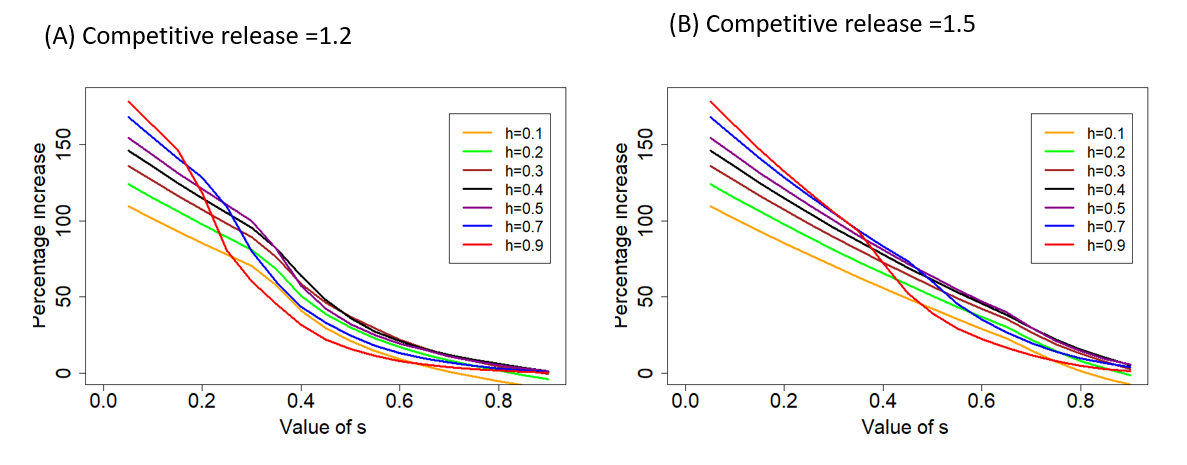

Supplement: jkad231_Supplementary_Data [file jkad231_supplementary_data.zip › G3-2023-404216R1_Supplemental_File_S1.docx]
