# Supplementary material for: The impact of Fisher's reproductive compensation on raising equilibrium frequencies of semidominant, nonlethal mutations under mutation/selection balance
Source: G3 (Bethesda). 2023 Nov 16;14(1):jkad231. doi: 10.1093/g3journal/jkad231 (PMC10755198; doi:10.1093/g3journal/jkad231)
Supplement: jkad231_Supplementary_Data [file jkad231_supplementary_data.zip › G3-2023-404216R1_Supplemental_File_S2.docx]

***Supplementary Material, S2: explanation for why simulation results did not fully recover the standard algebraic results for semi-dominant sex-linked mutations (equation 7 of main text).***

Simulations of sex-linked mutations recovered the std algebraic result (Equation 7 in main text) when *h* was large and/ or *s* small, but increasingly diverged by up to 18% as *h* decreased and/or *s* increased, see Fig. S2.1. This section discusses the likely cause of this discrepancy.

Derivation of Equation 7 (main text) rested on two assumptions that are the likely cause of this divergence i.e. that (i) “with weak selection, we can again use mean frequencies over males and females to represent the state of the population” (Charlesworth and Charlesworth 2010, page 98) and, (ii) consequently this use of mean frequencies generates Hardy-Weinberg frequencies of offspring. Notably, the term “weak selection” is not defined. I argue that the simulation results are the correct ones to use when evaluating the impact of fRC over the parameter space investigated in this work for several reasons.

(1) The simulations do not require the simplifying assumptions made in deriving the algebraic results. We can quantify the difference in selection between sexes as the ratio of mutation fitness i.e. (1-*s*) for males compared to (1-*hs*) for females. The smaller the ratio the less valid the assumption of equality of allele frequencies in males and female after selection. The ratio become smaller in parameter space where the simulated and algebraic results diverge (Fig. S2.2)

(2) In the preprint that precedes this paper (Hastings 2021), I used a methodology that tracked gametes frequencies and allowed a second round of random mating each generation, and tracked mean mutation frequency across sexes to equilibrium. Those simulations were less biologically realistic but reflect the assumptions made in the algebraic derivation of Equation 7. The results using that that methodology recover the algebraic results, implying the divergence is due to the assumptions in deriving Equation 7.

(3) The simulated results recover the other standard results (equations 8 and 9 of main text) suggesting their accuracy.

(4) The (usually unspoken) suspicion of simulated results is that a bug has crept into the code. There is no guarantee this has not occurred here but I list the checks made while running the code

- If s=0 then the diploid frequencies within broods should sum to unity (this checks there were no bugs in the mutation process).
- If s=0 and µ>0 then mutation should spread to fixation.
- If s>1 and µ=0 then mutation should be lost.
- If s=µ=0 then allele frequency should not change over the course of simulations
- There were various internal checks made each generation when running the simulations e.g. that the sum of mating frequencies is 1 etc

**Fig. S2.1**. Deviation between simulated and algebraic results for sex-linked semi-dominant mutations.


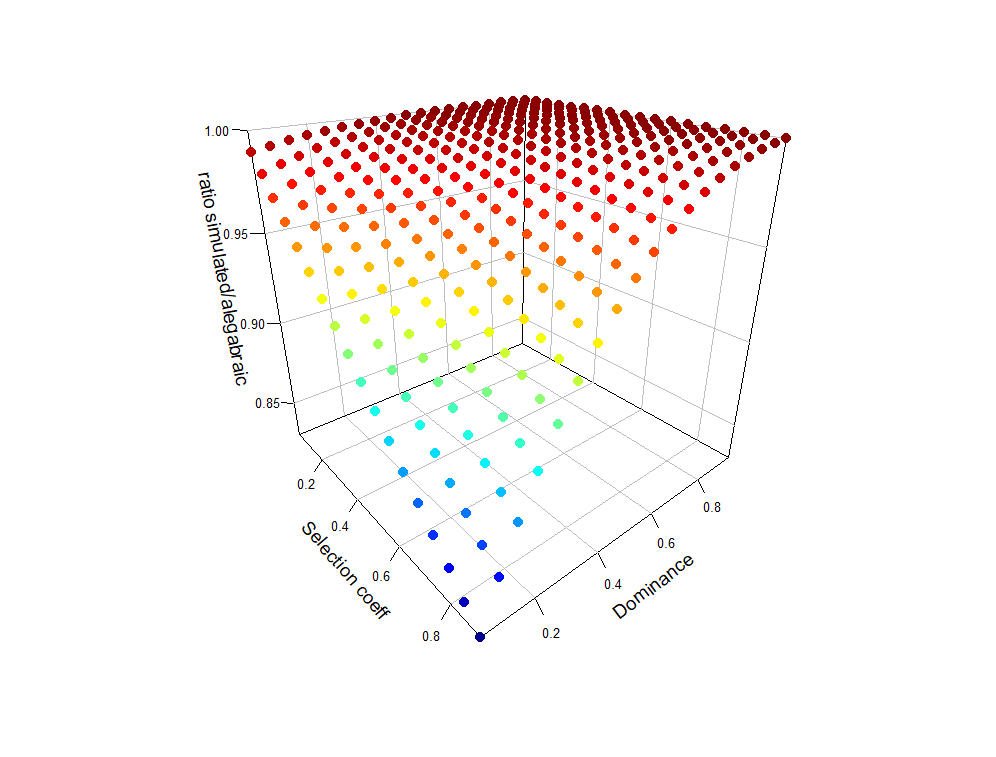


**Fig. S2.2.** How sex differences in intensity of selection against mutation varies with *h* and *s*. Sex differences were quantified as (1-*s*)/(1-*hs*)


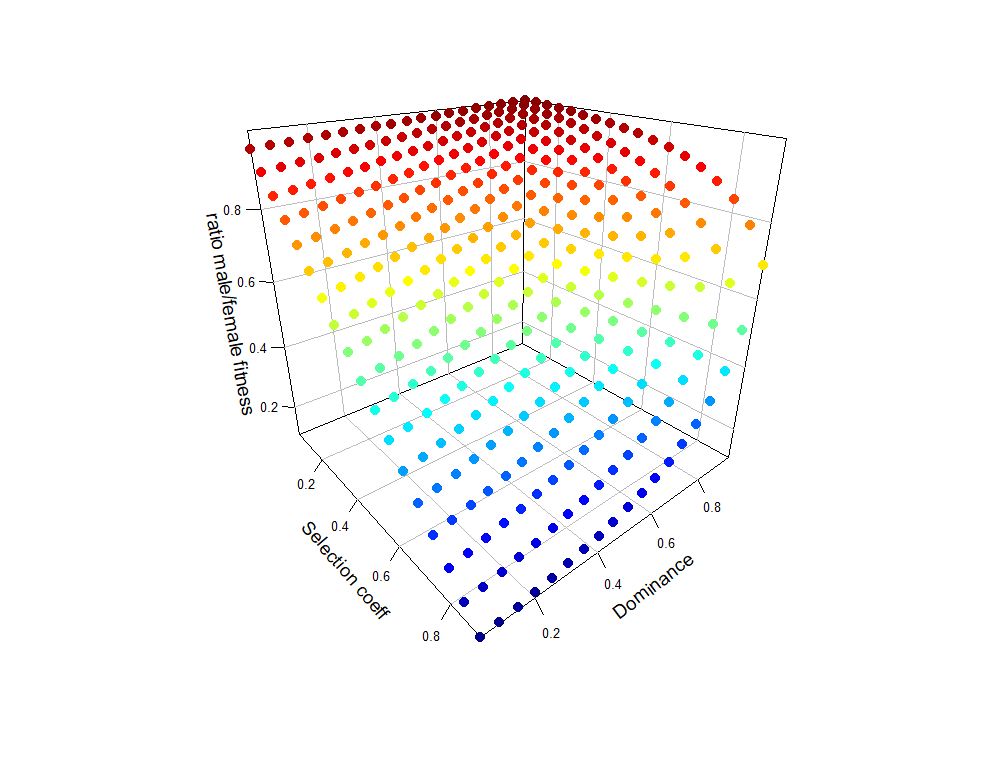


Charlesworth B, Charlesworth D. Elements of evolutionary genetics. Colorado: Roberts and company; 2010.

Hastings IM. The impact of Fisher’s Reproductive Compensation on raising equilibrium frequencies of semi-dominant, non-lethal mutations under mutation/selection balance. bioRxiv**:** 2021; 2011.2019.469230.
